# Colibactin genes are highly prevalent in the developing infant gut microbiome

**DOI:** 10.1080/19490976.2025.2604874

**Published:** 2025-12-17

**Authors:** Shira Levy, Kathryn E. McCauley, Rachel Strength, Emily S. Robbins, Qing Chen, Sivaranjani Namasivyam, George Maxwell, Suchitra K. Hourigan

**Affiliations:** a Clinical Microbiome Unit, Laboratory of Host Immunity and Microbiome, Division of Intramural Research, National Institute of Allergy and Infectious Disease, National Institute of Health, Bethesda, MD, USA; b Bioinformatics and Computational Biosciences Branch, National Institute of Allergy and Infectious Diseases, National Institutes of Health, Bethesda, MD, USA; c Women’s Service Line, Inova Health System, Falls Church, VA, USA

**Keywords:** Microbiota, neonate, neonatal intensive care unit, antibiotics, colorectal cancer

## Abstract

Early-life exposure to colibactin-producing *pks*+ gut bacteria is hypothesized to imprint mutations on the colorectal epithelium, increasing the risk of colorectal cancer later in life. We demonstrate an extremely high prevalence of *pks+*  bacteria (>50% of infants) during the first 2 y of life, suggesting carriage is likely normal during early-life microbiome development. Further investigation is required to understand the circumstances in which carriage can lead to mutagenesis.

## Main

Research has established that mutation signatures caused by colibactin, which is produced by bacteria carrying the *pks* pathogenicity island, contribute to the development of colorectal cancer (CRC).^1^^,^^2^ Recently it was found that these mutation signatures had higher mutation loads in countries with higher CRC incidence rates and were also enriched in early-onset CRC.^3^ It was hypothesized that early-life exposure to colibactin-producing *pks*+ gut bacteria may cause imprinting of mutations on the colorectal epithelium, as *pks*+ bacteria was not found at the time of tumor sequencing.^3^ While it is known that in the first few years of life gut microbiome development plays a critical role in immune system education, with disruptions in this critical window potentially having long lasting health impacts, the prevalence, dynamics and associated factors of *pks*+ gut bacteria carriage in early-life is unknown.

**Figure 1.**
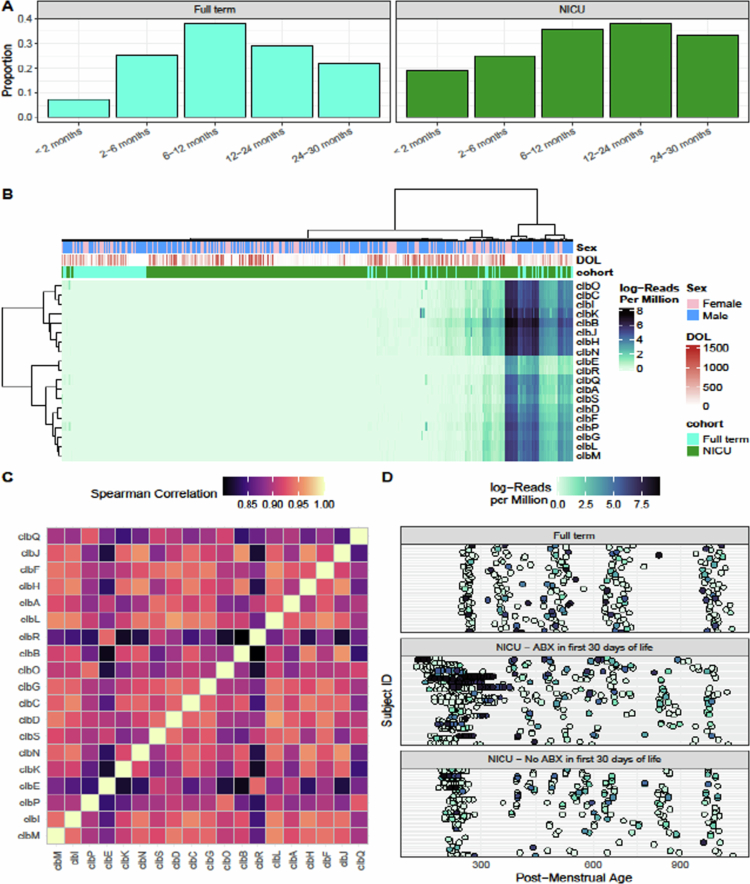
(A) Proportion of infants with pks+  samples. See the supplemental methods for information on the proportion calculations. (B) Heatmap of colibactin gene abundance as defined by log-transformed reads per million. Columns are annotated with infant sex (blue are males and pink are females), days of life (light to dark red), and cohort (full-term in teal or NICU in green). (C) Heatmap of Spearman correlations between each pair of colibactin genes; genes are arranged with Ward D2 clustering. (D) Colibactin gene abundance across samples; subjects are stratified into three groups based on cohort and antibiotic exposure; samples are arranged by post-menstrual age in days. Abbreviations: ABX: antibiotics; DOL: days of life.

To understand the potential role of colibactin in colorectal cancer development, it's essential to investigate the pks+ gut bacteria carriage in the early-life microbiome. We interrogated shotgun metagenomic sequencing of serial stool samples from the United States over the first 2 y of life from full-term infants (*N* = 55 infants, *n* = 275 samples) and infants who were predominantly preterm and spent time in the neonatal intensive care unit (NICU) (*N* = 128 infants, *n* = 877 samples) (cohort characteristics in the Supplementary Information). Using a previously established definition, pks+ samples had at least one read in >8 colibactin genes,^3^ with a median of 101 (IQR: 1116) reads per million (RPM) among pks+ samples, and a uniform distribution on the log scale; no sample contributed only one read across all eight genes towards pks-positivity. In our study, 31/55 (56%) of full-term infants and 85/128 (66%) of NICU infants had at least 1 *pks*+ sample in the first 2 y of life. In the full-term cohort, the prevalence peaked at 6–12 months of age, with 38% of infants being positive, and then declined over time (Figure 1A). Among NICU infants, the prevalence was significantly higher than that among full-term infants at 2 months (OR = 3.02, *P* = 0.047), but also trended higher after 12 months (Figure 1A). In the full-term cohort, the decrease in positivity appears to be more robust than the NICU cohort (Figure 1A). This may be due to the NICU cohort being comprised of primarily preterm infants who often have delayed microbiome maturation in comparison to full-term infants (Supplementary Information). As the initial rates of positivity were higher in the NICU cohort, we also anticipate that if followed over a greater period of time, the rates would similarly decrease. In the only other study to our knowledge that looked at infant carriage of colibactin positive bacteria up to 1 month of life in Japan, high rates of *pks*+ (31%) were also observed.^7^ However, it is possible that they may have underestimated the carriage rates in early life because of only assessing carriage up to 1 month, as our data show peak carriage rates at 6–12 months (full-term) and 12–24 months (NICU) of age (Figure 1A). Our longitudinal samples provide a more comprehensive understanding of the carriage rates of colibactin-carrying bacteria in early life. Given the high prevalence of *pks*+ in the first 2 y of life, which is greater than the prevalence of CRC in industrialized countries,^3^ we hypothesize that carriage of colibactin-producing bacteria may be part of the normal developing infant microbiome without adverse effects in most circumstances. Certainly, this is the case for toxigenic *Clostridioides difficile*, a potential carcinogenic bacterium and pathobiont, with very high prevalence rates in infancy without detrimental effects.^8^ Indeed, in the full-term cohort where data was available, toxigenic *Clostridioides difficile* carriage was associated with significantly increased colibactin gene abundance (Generalized Estimating Equations (GEE) *β* = 1.30, *P* < 0.001). However, considering evidence showing the strong contribution of *pks+* to CRC, firm conclusions cannot be drawn at this time regarding the adverse effects of colibactin gene carriage in infancy, especially in individuals more prone to the development of CRC or with as yet unspecified environmental conditions.^1^^,^^2^ Additional research is needed to determine the timing and conditions under which *pks+* carriage leads to genetic mutation.

We next examined colibactin gene abundance, a measure better suited towards shotgun metagenomics, where colibactin genes were detected in 491/1152 (43%) samples from infants, with a mean of 342 RPM across all samples (Figure 1B). Notably, individual colibactin genes were highly correlated with each other (Spearman correlations > 0.8, Figure 1C), justifying the utilization of the sum of all colibactin genes moving forward in further analyses. We next established the longitudinal abundance of colibactin gene carrying bacteria (Figure 1D). A pattern was observed of very early-life carriage in NICU infants, predominantly in those who had received antibiotics in the first 30 d of life compared to the full-term cohort, who did not receive early-life antibiotics (Figure 1D, middle panel, GEE day of life by cohort interaction *p* = 0.0485). Antibiotics are known to disrupt the balance of the gut microbiome, frequently leading to a pro-inflammatory environment that may enable the overgrowth of colibactin gene carrying bacteria. Consequently, antibiotic use in early life can influence which microbes colonize the infant gut. However, it remains unclear whether this is due to altered colonization resistance, changes in the gut environment, or modifications in gut barrier integrity.^9^

**Figure 2.**
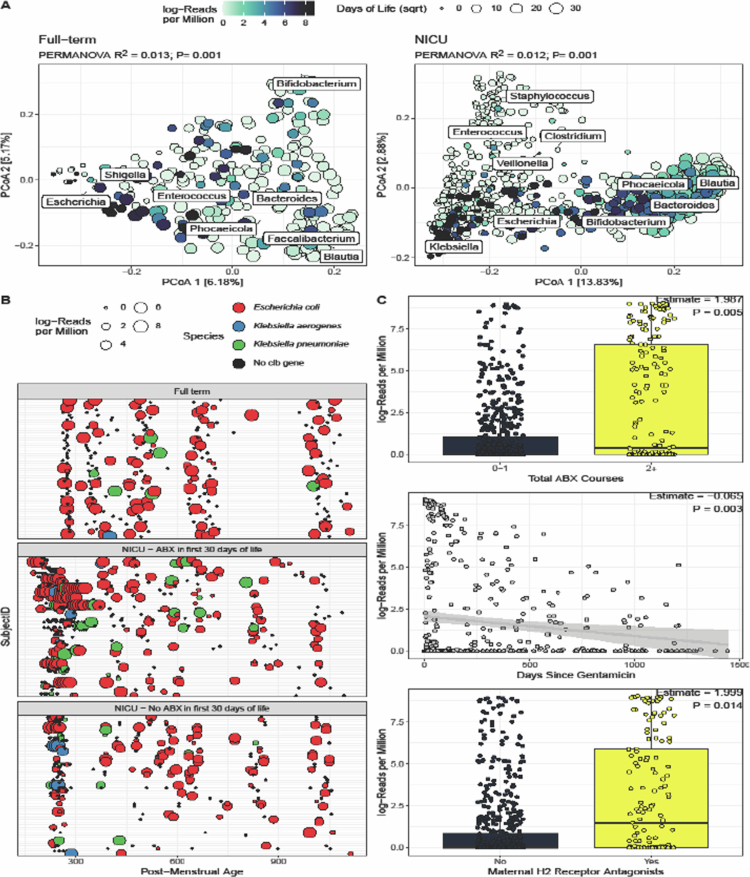
(A) Colibactin gene abundance compared to microbiome composition of the full-term (left) and NICU cohorts (right). Points are colored by colibactin gene abundance and sized by days of life (square-root transformed). Biplot indicates the influence of the most dominant genera on community composition. (B) Bacterial host of colibactin genes across samples and time points. The samples for which the colibactin gene was not identified are represented in black; the point size indicates the abundance of colibactin in the sample. Consistent with Figure 1C, the samples were stratified by cohort and antibiotic exposure. (C) In the NICU, the abundance of colibactin correlated with three variables: total antibiotic courses, days since gentamicin, and maternal H2 receptor antagonist intake.

Our analysis of the microbiome factors associated with colibactin abundance revealed no differences in alpha diversity; however, differences in microbiome composition (Canberra distance, Figure 2A) were detected in both the full-term (PERMANOVA R^2^ = 0.013, *P* = 0.001) and NICU (R^2^ = 0.012, *P* = 0.001) cohorts. In the full-term cohort, *Escherichia* was the microbial genus associated with increased colibactin abundance while microbiota enriched with *Bifidobacterium*, *Bacteroides, Fecalibacterium* and *Blautia* exhibited limited colibactin abundance. In the NICU cohort, *Klebsiella* was the microbial genus associated with higher colibactin abundance while *Staphylococus* and *Enterococcus* enriched microbiomes exhibited limited colibactin abundance.

We further investigated the specific species that carried colibactin genes, and *Escherichia coli* was the dominant species in both cohorts (Figure 2B). However, infants in the NICU cohort who received antibiotics in the first 30 d of life had higher odds of having colibactin contained in *Klebsiella pneumoniae* compared to the full-term cohort (OR = 3.49, *P* = 0.016). Notably, while most studies have investigated the role of colibactin carrying *Escherichia coli* in CRC, colibactin carrying *Klebsiella pneumoniae* may also play a role in CRC progression.^10^

We next explored the potential source of transmission of colibactin genes. The limited NICU parental samples exhibited lower *pks+* (4/18, 22%) and low levels of detection (mean of 11.5 RPM across all genes). As the NICU cohort parental samples did not exhibit high levels of colibactin genes and the NICU cohort was primarily composed of cesarean section delivery (Supplementary Information), we anticipate that the parents were not the primary source of transmission. This contrasts with previous literature, which suggests that the colibactin genes are vertically transmitted at birth.^7^ A key difference in our study is the inclusion of the large NICU cohort; however, even in our full-term cohort, there was no association with colibactin gene abundance and vaginal delivery. We then hypothesized that these genes may be sourced from the NICU environment, as we have previously shown colonization in infants from the NICU environment.^11^ However, only 3/86 (3%) of the environmental samples had more than 5 RPM for at least 8 colibactin genes, suggesting that this was an unlikely source for these genes. The source of these genes is currently unknown, and additional parental samples from every infant may be beneficial for elucidating the source.

We next examined the clinical factors associated with colibactin gene abundance. In the full-term cohort, after FDR correction, no clinical or demographic factors were significantly associated with colibactin gene abundance, including delivery mode, receiving breast milk and antibiotic use. In the NICU cohort, carriage in the first 6 months of life was positively associated with the total number of courses of antibiotics infants received within the NICU (*P* = 0.001, Figure 2C), with the most common antibiotics received being gentamicin and ampicillin within the first 48 h of life. Additionally, the days since receiving gentamicin were negatively correlated with colibactin abundance (*P* < 0.001, Figure 2C). Accordingly, systematic reviews have shown an increased risk of CRC with any antibiotic use and higher levels of antibiotic exposure.^12^^,^^13^ A lack of association with antibiotic use in the full-term cohort may be due to the cohort consisting of generally healthy infants with minimal antibiotic use (Supplementary Information). In the NICU cohort, the colibactin gene abundance was also higher in infants whose mothers had received H2 receptor antagonists during pregnancy (*P* = 0.014, Figure 2C). While gastric acid suppressing mediations are known to alter the gut microbiome, theoretically making it more permissive to colibactin carrying bacteria, there is no direct evidence for this association or how mothers taking the medication affect the infants’ risk of carriage.^13^

Our research revealed that *pks*+ bacteria were highly prevalent (>50% of infants) in the developing early-life gut microbiome in the first 2 y of life. This far surpasses the rates of CRC, even in industrialized countries.^3^ Given the very high prevalence, we hypothesize that this is likely a part of the normal developing gut microbiome, without harmful effects in most circumstances. Further exploration is warranted to assess when carriage of these bacteria in early-life leads to pathogenic colibactin expression. To address this question, we suggest conducting lifespan cohort studies with serial sampling and genetic susceptibility determination, intestinal organoid models and transplantation of infant stool into murine models. In addition, the currently unknown prevalence rates of *pks*+ bacteria carriage in early-life should be investigated in non-industrialized countries where CRC and early onset CRC rates are lower.^3^^,^^14^ If conditions are found where early-life carriage of *pks*+ bacteria can cause imprinting of mutations associated with later CRC, modulation of the gut microbiome in early-life may represent a potential therapeutic target, given the microbiome compositional changes associated with carriage.

## Supplementary methods

### Subjects and sample collection

#### Full-term cohort

Mothers were enrolled prenatally with informed consent in a longitudinal, prospective cohort study titled “The First 1000 Days of Life and Beyond” within the Inova Health System. All experimental protocols were approved by the Inova Health System and WCG Institutional Review Board (Inova protocol #15-1804, WCG protocol #20120204). In brief, serial stool samples were collected from infants within the first 2 d of life (meconium) and at approximately 2 m, 6 m, 12 m, and 24 m of age as previously described.^15^ All samples were collected using previously validated methods and stored at −80 °C until analysis.^16^ Maternal demographic and clinical information, including mode of delivery and use of prenatal antibiotics and peripartum antibiotics was collected via questionnaires and electronic medical records review. Infant data such as gestational age at delivery, use of antibiotics, and medical history were also collected from parents through questionnaires.

#### Neonatal intensive care unit (NICU) cohort

Neonates who spent time in the Inova Fairfax Neonatal Intensive Care Unit (NICU) were enrolled in an observational longitudinal microbiome cohort study, “Neonatal Intestinal Microbiome: Impact on Infant and Early Childhood Health and Disease”, as previously described.^17^ The study was approved by the Institutional Review Board (WCG IRB 1300205), and parental informed consent was obtained. Neonates were enrolled within the first week of life and had an anticipated stay in the NICU of >5 d. In the NICU, stool samples were collected for microbiome analysis up to twice a week and stored at −80 °C until analysis. Detailed demographic and clinical data were collected, including delivery mode, gestational age, maternal peripartum antibiotic use and infant antibiotics.

After discharge from the NICU, follow-up surveys reporting health, illnesses, diet and a variety of exposures were collected approximately every 3–6 months until approximately 2 y of age (corrected for gestational age), accompanied by a stool sample collected by previously validated methods.^18^ For the present study, neonates from the larger cohort were included if they had at least 1 stool sample from before 1 month of life, and at least 2 stool samples overall were collected.

Environmental swabs were collected with a sterile cotton swab moistened with sterile saline and rolled over the area in a standardized manner from different areas in the NICU where the infants were hospitalized, including the wash sink at the nurses’ station, the desk at the nurses’ station, the light switch and the foam hand sanitizer pump as previously described.^17^^,^^19^

### DNA extraction

DNA was extracted from fecal samples in two stages as previously described.^20^ First, approximately 50 mg of fecal material and 650 μL MBL lysis buffer from the PowerMicrobiome DNA/RNA EP Kit (Qiagen) were added to Lysis Matrix E (LME) tubes (MP Biomedicals). LME tubes were transferred to a Precelleys 24 Tissue Homogenizer (Bertin Technologies), and fecal samples were homogenized, centrifuged, with the resulting supernatant was transferred to a deep-well 96-well plate. The second stage consisted of DNA isolation from the above supernatant using the MagAttract PowerMicrobiome DNA/RNA EP Kit (Qiagen) on an automated liquid handling system as detailed by the manufacturer (Eppendorf).

### Shotgun metagenomic sequencing

The total gene content of the microbiome was assessed through shotgun metagenomic sequencing as previously described.^20^ Metagenomic libraries were constructed from 100 ng of DNA as starting material using the Illumina DNA Prep Kit. Illumina DNA/RNA UD indices were used to add sample-specific sequencing indices to both ends of the libraries. An Agilent 4200 TapeStation system with High Sensitivity D5000 ScreenTape (Agilent Technologies, Inc.) was used to verify the quality and assess the final library size. A positive control (MSA-2002 20 Strain Even Mix Whole Cell Material (ATCC)) and a buffer extraction negative control were included. Metagenomic libraries were normalized and pooled at an equimolar concentration. Final pools were sequenced on the NovaSeq platformn using a paired-end (150 × 150) producing 2 × 150 bp paired-end reads (Illumina, Inc).

#### Metagenomic analysis

Extant metagenomic reads from the two cohorts underwent quality trimming with fastp v0.23.4 and human reads were filtered with Kraken2 v2.1.3 against the human genome using Whole Genome Shotgun Assembly (WGSA2) from the Nephele platform. Environmental samples were trimmed with fastp and decontaminated for human reads with Ganon2, as they were single-end and not amenable to the WGSA2 pipeline.^24^ Reads from paired-end samples were assembled into contiguous sequences with metaSPAdes v4.0.0 in Nephele, and genes were predicted using Prodigal v2.6.3.^25^^,^^26^

All non-human reads were separately aligned to the IHE3034 genome (RefSeq assembly: GCF_000025745.1) using bbmap v39.01, and reads per million for each of the 19 colibactin genes were obtained.^27^ In order to determine the bacteria containing colibactin, genes identified by Prodigal were aligned against the set of colibactin genes in the reference genome with bbmap. Those alignments were taxonomically annotated with Ganon2 using a relative cutoff of 0.2.^24^ The database contained NCBI complete genomes built on June 25, 2025, with default parameters.

#### Statistical analysis

The prevalence of pks+ samples was calculated within the age ranges used by visits for the full-term cohort; a 20-d buffer prior to the later sample collection timepoint was used to ensure that participants early in their visit were grouped correctly. All samples from each time period were used; for each time period, the number of positive samples was divided by the total number of samples available for a subject to account for repeated sampling in the NICU cohort. These subject-level proportions were summed to create a weighted proportion at each timepoint. Permutational analysis of variance (PERMANOVA) was performed with adonis2 in the vegan package v2.7-2.^28^ Correlations between colibactin genes were performed using Spearman correlation, and hierarchically clustered using the Ward D2 method. Generalized Estimating Equations (GEE) from the glmtoolbox package v0.1.12 were used to compare longitudinal colibactin abundance with cohort factors, including *Clostridioides difficile* detection, using an exchangeable correlation structure.^29^

## Supplementary Material

Supplementary material **Supplemental Table 1:** Description of demographic and clinical characteristics of the full-term and NICU Cohorts.

## Data Availability

Data are publicly available from the Sequence Read Archive for the full-term cohort under the accession PRJNA988496, and for the NICU cohort under the accession PRJNA1280936. Environmental samples are available from PRJNA417283. This paper does not report original code.

## References

[1] Chen B, Ramazzotti D, Heide T, Spiteri I, Fernandez-Mateos J, James C, Magnani L, Graham TA, Sottoriva A. Contribution of pks(+) *E. coli* mutations to colorectal carcinogenesis. Nat Commun. 2023;14:7827. doi: 10.1038/s41467-023-43329-5. 38030613 PMC10687070

[2] Pleguezuelos-Manzano C, Puschhof J, Rosendahl Huber A, van Hoeck A, Wood HM, Nomburg J, Gurjao C, Manders F, Dalmasso G, Stege PB, et al. Mutational signature in colorectal cancer caused by genotoxic pks(+) *E. coli*. Nature. 2020;580:269–273. doi: 10.1038/s41586-020-2080-8. 32106218 PMC8142898

[3] Díaz-Gay M, dos Santos W, Moody S, Kazachkova M, Abbasi A, Steele CD, Vangara R, Senkin S, Wang J, Fitzgerald S, et al. Geographic and age variations in mutational processes in colorectal cancer. Nature. 2025;643:230–240. doi: 10.1038/s41586-025-09025-8. 40267983 PMC12221974

[4] Gensollen T, Iyer SS, Kasper DL, Blumberg RS. How colonization by microbiota in early life shapes the immune system. Science. 2016;352:539–544. doi: 10.1126/science.aad9378. 27126036 PMC5050524

[5] Yatsunenko T, Rey FE, Manary MJ, Trehan I, Dominguez-Bello MG, Contreras M, Magris M, Hidalgo G, Baldassano RN, Anokhin AP, et al. Human gut microbiome viewed across age and geography. Nature. 2012;486:222–227. doi: 10.1038/nature11053. 22699611 PMC3376388

[6] Cox LM, Yamanishi S, Sohn J, Alekseyenko AV, Leung JM, Cho I, Kim SG, Li H, Gao Z, Mahana D, et al. Altering the intestinal microbiota during a critical developmental window has lasting metabolic consequences. Cell. 2014;158:705–721. doi: 10.1016/j.cell.2014.05.052. 25126780 PMC4134513

[7] Tsunematsu Y, Hosomi K, Kunisawa J, Sato M, Shibuya N, Saito E, Murakami H, Yoshikawa Y, Iwashita Y, Miyoshi N, et al. Mother-to-infant transmission of the carcinogenic colibactin-producing bacteria. BMC Microbiol. 2021;21:235. doi: 10.1186/s12866-021-02292-1. 34429063 PMC8386082

[8] Mani J, Levy S, Angelova A, Hazrati S, Fassnacht R, Subramanian P, Richards T, Niederhuber JE, Maxwell GL, Hourigan SK. Epidemiological and microbiome associations of *Clostridioides difficile* carriage in infancy and early childhood. Gut Microbes. 2023;15:2203969. doi: 10.1080/19490976.2023.2203969. 37096914 PMC10132246

[9] Jans M, Vereecke L. Physiological drivers of pks+ *E. coli* in colorectal cancer. Trends Microbiol. 2025;33:1003–1017. doi: 10.1016/j.tim.2025.04.010. 40335416

[10] Kaur CP, Iyadorai T, Sears C, Roslani AC, Vadivelu J, Samudi C. Presence of polyketide synthase (PKS) gene and counterpart virulence determinants in *Klebsiella pneumoniae* strains enhances colorectal cancer progression in-vitro. Microorganisms. 2023;11:443. doi: 10.3390/microorganisms11020443. 36838407 PMC9965769

[11] Chaudhary PP, O'Laughlin B, Kumar PS, Dabdoub SM, Levy S, Myles IA, Hourigan SK. Vaginal delivery provides skin colonization resistance from environmental microbes in the NICU. Clin Transl Med. 2023;13:e1506. doi: 10.1002/ctm2.1506. 38058267 PMC10701179

[12] Liu YC, Tang X, Lang J, Qiu Y, Chen Y, Li X, Cao Y, Zhang C. Effects of antibiotic exposure on risks of colorectal tumors: a systematic review and meta-analysis. J Transl Med. 2025;23:682. doi: 10.1186/s12967-025-06727-5. 40533779 PMC12178024

[13] Aneke-Nash C, Yoon G, Du M, Liang P. Antibiotic use and colorectal neoplasia: a systematic review and meta-analysis. BMJ Open Gastroenterol. 2021;8:e000601. doi: 10.1136/bmjgast-2021-000601. PMC8174505 34083227

[14] Mäklin T, Taira A, Arredondo-Alonso S, Shao Y, Stratton MR, Lawley TD, Aaltonen LA, Corander J. Geographical variation in the incidence of colorectal cancer and urinary tract cancer is associated with population exposure to colibactin-producing *Escherichia coli*. Lancet Microbe. 2025;6:101015. doi: 10.1016/j.lanmic.2024.101015. 39644909

[15] Subramanian P, Romero-Soto HN, Stern DB, Maxwell GL, Levy S, Hourigan SK. Delivery mode impacts gut bacteriophage colonization during infancy. Gut Microbes Rep. 2025;2:2464631. doi: 10.1080/29933935.2025.2464631. 40823537 PMC12352455

[16] McDonald D, Hyde E, Debelius JW, Morton JT, Gonzalez A, Ackermann G, Aksenov AA, Behsaz B, Brennan C, Chen Y, et al. American Gut: an open platform for citizen science microbiome research. mSystems. 2018;3(3). doi: 10.1128/mSystems.00031-18. PMC5954204 29795809

[17] Hourigan SK, Subramanian P, Hasan NA, Ta A, Klein E, Chettout N, Huddleston K, Deopujari V, Levy S, Baveja R, et al. Comparison of infant gut and skin microbiota, resistome and virulome between Neonatal Intensive Care Unit (NICU) environments. Front Microbiol. 2018;9:1361. doi: 10.3389/fmicb.2018.01361. 29988506 PMC6026636

[18] Wong WSW, Clemency N, Klein E, Provenzano M, Iyer R, Niederhuber JE, Hourigan SK. Collection of non-meconium stool on fecal occult blood cards is an effective method for fecal microbiota studies in infants. Microbiome. 2017;5:114. doi: 10.1186/s40168-017-0333-z. 28870234 PMC5583988

[19] Chaudhary PP, Myles IA, Zeldin J, Dabdoub S, Deopujari V, Baveja R, Baker R, Bengtson S, Sutton A, Levy S, et al. Shotgun metagenomic sequencing on skin microbiome indicates dysbiosis exists prior to the onset of atopic dermatitis. Allergy. 2023;78:2724–2731. doi: 10.1111/all.15806. 37422700 PMC10543534

[20] Akagbosu CO, McCauley KE, Namasivayam S, Romero-Soto HN, O’Brien W, Bacorn M, Bohrnsen E, Schwarz B, Mistry S, Burns AS, et al. Gut microbiome shifts in adolescents after sleeve gastrectomy with increased oral-associated taxa and pro-inflammatory potential. Gut Microbes. 2025;17:2467833. doi: 10.1080/19490976.2025.2467833. 39971742 PMC11845021

[21] Chen S, Zhou Y, Chen Y, Gu J. fastp: an ultra-fast all-in-one FASTQ preprocessor. Bioinformatics. 2018;34:i884–i890. doi: 10.1093/bioinformatics/bty560. 30423086 PMC6129281

[22] Wood DE, Lu J, Langmead B. Improved metagenomic analysis with Kraken 2. Genome Biol. 2019;20:257. doi: 10.1186/s13059-019-1891-0. 31779668 PMC6883579

[23] Weber N, Liou D, Dommer J, MacMenamin P, Quiñones M, Misner I, Oler AJ, Wan J, Kim L, Coakley McCarthy M, et al. Nephele: a cloud platform for simplified, standardized and reproducible microbiome data analysis. Bioinformatics. 2018;34:1411–1413. doi: 10.1093/bioinformatics/btx617. 29028892 PMC5905584

[24] Piro VC, Reinert K. ganon2: up-to-date and scalable metagenomics analysis. NAR Genom Bioinform. 2025;7:lqaf094. doi: 10.1093/nargab/lqaf094. 40677913 PMC12267982

[25] Nurk S, Meleshko D, Korobeynikov A, Pevzner PA. metaSPAdes: a new versatile metagenomic assembler. Genome Res. 2017;27:824–834. doi: 10.1101/gr.213959.116. 28298430 PMC5411777

[26] Hyatt D, Chen G, LoCascio PF, Land ML, Larimer FW, Hauser LJ. Prodigal: prokaryotic gene recognition and translation initiation site identification. BMC Bioinformatics. 2010;11:119. doi: 10.1186/1471-2105-11-119. 20211023 PMC2848648

[27] Berkeley B. 2014. (Ernest Orlando Lawrence Berkeley National Laboratory, Berkeley, CA (US), United States).

[28] Oksanen JBF, Kindt R, Legendre P, Minchin P, O'Hara B, Simpson G, Solymos P, Stevens H, Wagner H. Vegan: Community Ecology Package. 2022.

[29] https://CRAN.R-project.org/package=glmtoolbox.

[30] Levy S, McCauley K, Strength R, Robbins E, Chen Q, Namasivayam S, Maxwell GL, Hourigan SK. Colibactin genes are highly prevalent in the developing infant gut microbiome. medRxiv. 2025. doi: 10.1101/2025.08.12.25333511. PMC12716031 41405404

